# Risk factors for early-onset colorectal cancer: systematic review and meta-analysis

**DOI:** 10.3389/fonc.2023.1132306

**Published:** 2023-05-05

**Authors:** Hongmei Hua, Qiuping Jiang, Pan Sun, Xing Xu

**Affiliations:** Department of Nursing, The Second Affiliated Hospital of Zhejiang University School of Medicine, Hangzhou, Zhejiang, China

**Keywords:** colorectal cancer, early-onset, risk factors, demographics, chronic conditions, lifestyle factors, environmental factors

## Abstract

**Background:**

The incidence of early-onset colorectal cancer (EOCRC), which means colorectal cancer diagnosed in patients under 50 years, has been increasing around the world. However, the etiology remains unclear. This study aims to identify risk factors for EOCRC.

**Methods:**

This systematic review was conducted in PubMed, Embase, Scopus, and Cochrane Library databases from inception to November 25, 2022. We examined risk factors for EOCRC, including demographic factors, chronic conditions, and lifestyle behaviors or environmental factors. Random-effects/fixed-effects meta-analysis was adopted to combine effect estimates from published data. Study quality was evaluated with the Newcastle-Ottawa Scale (NOS). Statistical analysis was performed Revman5.3. Studies not suitable for the meta-analysis were analyzed by a systematic review.

**Results:**

A total of 36 studies were identified for this review, and 30 studies were included in the meta-analysis. Significant risk factors for EOCRC included male (OR=1.20; 95% CI, 1.08-1.33), Caucasian (OR=1.44; 95% CI, 1.15-1.80), a family history of CRC (OR=5.90; 95% CI, 3.67-9.48), inflammatory bowel disease (OR=4.43; 95% CI, 4.05-4.84), obesity (OR=1.52; 95%CI, 1.20-1.91), overweight (OR=1.18; 95% CI, 1.12-1.25), triglycerides (OR=1.12; 95% CI, 1, 08-1.18), hypertension (OR=1.16; 95% CI, 1.12-1.21), metabolic syndrome (OR=1.29; 95% CI, 1.15-1.45), smoking (OR=1.44; 95% CI, 1.10-1.88), alcohol consumption (OR=1.41; 95% CI, 1.22-1.62), a sedentary lifestyle (OR=1.24; 95% CI, 1.05-1.46), red meat (OR=1.10; 95% CI, 1.04-1.16), processed meat (OR=1.53; 95% CI, 1.13-2.06), Western dietary patterns (OR=1.43; 95% CI, 1.18-1.73) and sugar-sweetened beverages (OR=1.55; 95% CI, 1.23-1.95). However, no statistical differences were found for hyperlipidemia and hyperglycemia. Vitamin D may be a protective factor (OR=0.72; 95% CI, 0.56-0.92). There was considerable heterogeneity among studies (I^2^>60%).

**Conclusions:**

The study provides an overview of the etiology and risk factors of EOCRC. Current evidence can provide baseline data for risk prediction models specific to EOCRC and risk-tailored screening strategies.

## Introduction

Colorectal cancer (CRC) is the third most common cancer worldwide and the second most common cause of cancer-related death (1). In recent decades, the number of cases of early-onset colorectal cancer (EOCRC), defined as CRC diagnosed before age 50 (2), has increased dramatically in the United States (3), Japan, Australia (4), Canada (5), China, the United Kingdom, and other countries (6). According to the current data, it is estimated that in the next ten years, the incidence rates of rectal cancer and colon cancer in adults aged 20-34 will increase by 90% and 124% respectively, while the incidence rate in adults aged 35-49 will increase by 27% and 46% respectively (7). Moreover, most young CRC patients have a later stage of disease at diagnosis, a higher risk of metastasis, and a poorer prognosis than elderly CRC patients, which highlights the need for the public and medical personnel to enhance their understanding of the disease (8).

Multiple risk factors have been identified, such as a family history of CRC, inflammatory bowel disease (IBD), sedentary behaviors, smoking, elevated body mass index (BMI), diabetes, and poor diet (9). However, the conclusions of different studies remain controversial, such as obesity, data from a prospective cohort study (10) and an observational study (11) showed that obesity during adolescence was associated with an increased incidence and mortality of EOCRC. A cross-sectional study (12) of local Songjiang District community residents in the city of Shanghai, east of China also found that obesity was an independent risk factor for early colorectal neoplasm. In contrast, Low et al. (13) found that obesity or overweight was a protective factor for EOCRC. The risk factors for EOCRC remain unclear. Identifying risk factors for EOCRC can inform primary prevention programs and targeted screening approaches for high-risk individuals.

To the best of our knowledge, O’Sullivan et al. (14) conducted a meta-analysis of risk factors for EOCRC and showed that smoking was not significantly associated with EOCRC. However, we found that some potentially important studies were not included in this study, such as a study (15) involving 8,873,080 people, which compared patients with EOCRC with healthy individuals under 50 and illustrated that smoking was significantly associated with the incidence rate of EOCRC (adjusted OR=2.675; P<0.001), which may affect the results. In addition, there were few studies included in the above meta-analysis, and the results may be biased. Carroll et al. (16) and Li et al. (17) only evaluated a single factor in their systematic reviews, and could not determine all the risk factors related to EOCRC. With the in-depth study of EOCRC in recent years, it is very important to identify the risk factors that may increase EOCRC through a comprehensive literature search.

To improve knowledge based on existing evidence and to address the limitations of previous reviews, we conducted a systematic review and meta-analysis to investigate risk factors for EOCRC. Our goal was to synthesize previous studies, which we hypothesized would be highly heterogeneous, under a uniform framework that can provide insights into development of new, evidence-based identification of risk factors associated with EOCRC, exploration of more effective prevention methods, and improved screening of high-risk populations.

## Methods

The protocol was registered with PROSPERO (CRD42022371340). We conducted this systematic review and meta-analysis according to the guidelines of Preferred Reporting Items for Systematic Reviews and Meta-Analyses (PRISMA) (18) and Meta-analysis of Observational Studies in Epidemiology recommendations (19).

### Search strategy

A literature search without any country restriction was performed to identify studies that described risk factors for EOCRC. Two researchers (QP J and HM H) independently searched PubMed (1950-present), Embase (1947-present), Scopus (1970-present), and the Cochrane Library (1995-present) from inception to November 25, 2022. The following keywords or terms were used to search: early-onset colorectal cancer, EOCRC, young colorectal cancer, risk factors, and risk. The complete search strategy is provided in the **Supplementary Material**. In addition, study references and study lists cited in articles related to the topic were browsed and manually searched to determine if any study had performed subgroup analyses in subjects younger than 50 years, further supplementing eligible studies for our study.

### Study inclusion and exclusion

The inclusion criteria were as follows: (1) study design: case-control or cohort studies, (2) studies of which full-text could be obtained, (3) studies reporting the risk factors related to EOCRC, (4) studies comparing patients with EOCRC and healthy individuals younger than 50 years, and (5) studies on patients with CRC diagnosed for the first time. The exclusion criteria were as follows: (1) studies comparing characteristics between EOCRC cases and late-onset cases, (2) studies on patients with advanced polyps, (3) studies not reporting effect estimates or from which effect estimates could not be obtained, or (4) studies not published in English.

### Study selection

According to the inclusion and exclusion criteria, literature screening and data extraction were completed independently by two researchers (QP J and X X) and then cross-checked. Any conflict was resolved by HM H.

### Risk of bias and quality assessment

Study quality was evaluated by two researchers (QP J and P S) using the Newcastle-Ottawa Scale (NOS) (20). The NOS is a quality scale that evaluates studies based on 3 broad categories: selection (maximum of four stars); comparability (maximum of two stars); and exposure/outcome (maximum of three stars). This scale has a total score of 9 stars, and a study with a score ≥6 stars is considered to have “good” quality. Differences of opinion during the evaluation were resolved through mutual discussion or consultation with a third researcher (HM H).

### Data extraction

We extracted data on characteristics of the included studies, including author, year of publication, country, study type, age at diagnosis of EOCRC, sample size, geographic location, participant sex, and population selection. We extracted information about the types of risk factors (demographic characteristics, lifestyle or environmental factors, clinical factors, comorbidities, reproductive factors, genetic factors), and measurement of risk factors (cutoff value for clinical factors, diagnosis of comorbidities), and extracted the referent category, effect estimates, 95% confidence intervals (CIs) for exposure categories of each risk factor. Risk factors could be obtained through patient self-reported or physician measurements.

### Statistical analysis

For the purposes of this study, the relative ratios and hazard ratios were treated as estimates of odds ratios (ORs). We performed log transformation on the extracted ORs and indirectly estimated their standard errors. As a risk factor was reported in at least 2 studies, we included these studies in meta-analyses. RevMan5.3 was used for statistical analysis. Cochrane Q-test and I^2^ test were used to evaluate heterogeneity. Fixed-effects models or random-effects models were selected according to the heterogeneity of test results. Sensitivity analysis was used to identify the source of heterogeneity. If more than 10 studies were included in the meta-analysis, funnel plots were used to assess the risk of publication bias for each risk factor. Studies not suitable for the meta-analysis were analyzed through a systematic review.

We used the BMI classification of the World Health Organization. Due to the different classifications of BMI in the included studies, we performed the meta-analysis using two methods: (1) making comparison between obese (BMI>30 kg/m^2^) and normal-weight subjects, and (2) making comparison between overweight (30≥BMI≥24.9 kg/m^2^) and normal-weight subjects. We also evaluated the effect of abdominal obesity on EOCRC. Glover et al. (15) did not define obesity and Kwak et al. (21) only compared individuals with BMI≥25 kg/m^2^ with the general population, so we did not include it in the analysis. For smoking, we pooled estimates of the effects of smokers (former and current) and never-smokers. For alcohol consumption, we pooled estimates of the effects of drinkers (past and current) and never-drinkers. We included dyslipidemia in the analysis, and a separate meta-analysis was conducted on triglyceride. In terms of diet, due to the limited number of studies, we only included red meat, processed meat, Western dietary patterns, sugar-sweetened beverages, and vitamin D for the meta-analysis. All *P* values were two-sided, and the significance level was set at 0.05.

## Results

**Figure 1:**
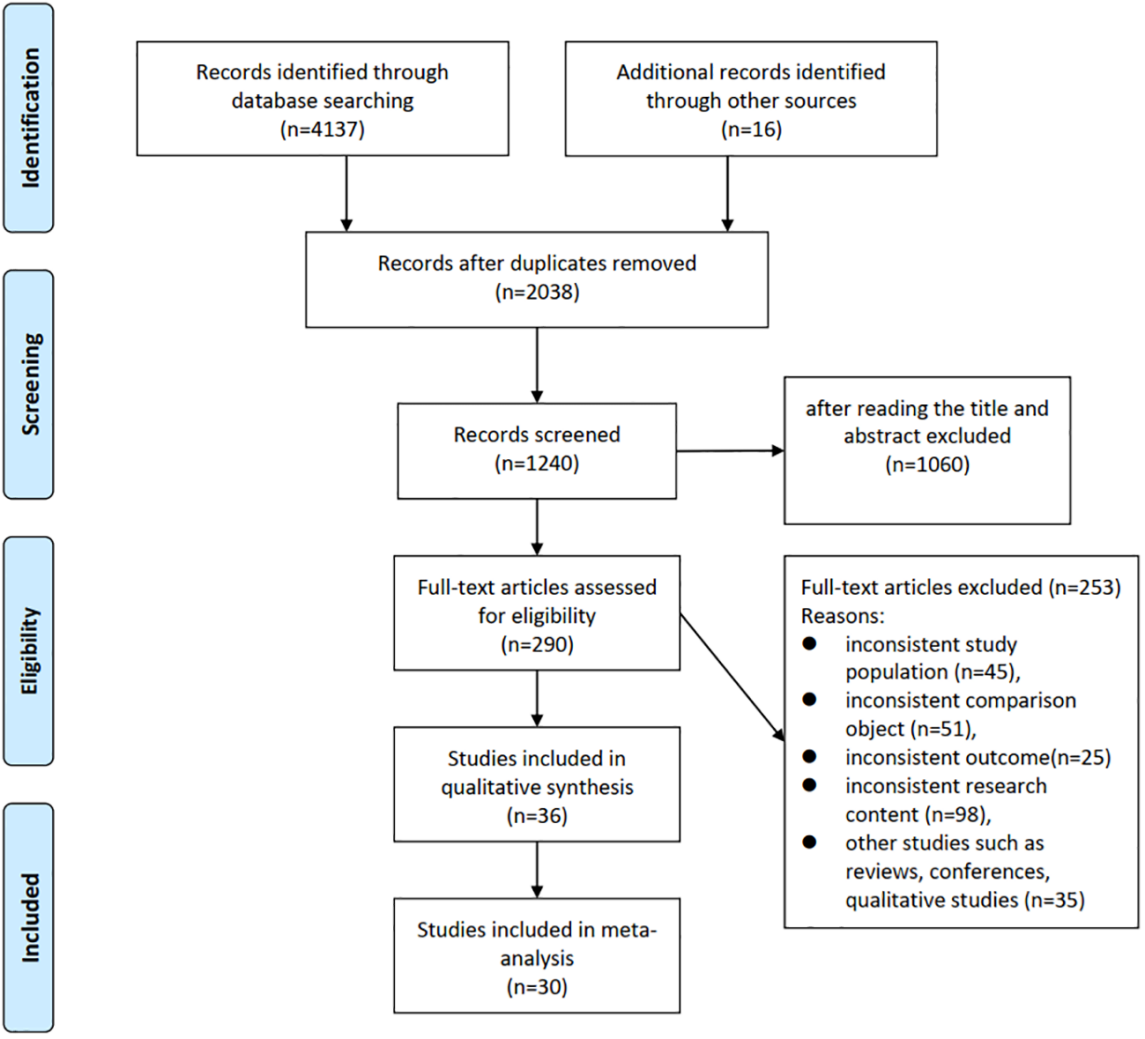
PRISMA flow diagram.

Initial searches identified 4137 studies, and 16 additional studies were identified from references. Subsequently, 2038 studies were obtained after removing duplicate literature, and 1060 were excluded after screening titles and abstracts. After full-text review, a total of 36 studies examining at least one risk factor for EOCRC were retained, and 30 of these studies were included in the meta-analysis (**Figure 1**).

Among the 36 studies (13, 15, 21–54), 19 were cohort studies and 17 were case-control studies, with 66312 participants. The year of publication ranged from 1989 to 2022, and studies in the last three years accounted for 56.76% (**Table 1**).

**Table 1:** Characteristics of all studies investigating risk factors for the development of early-onset (<50 years of age) colorectal cancer.

First Author (year)	Study Type	Age at Diagnosis of EOCRC	Sample Size (cases)	Location	Sex	Outcome	Risk Factors Identified	Population Selection
Puzzono 2022 (22)	case-control (2018–2021)	18-49	60	Italy	All	CRC	family history, processed meat, dairy products, smoking	third-level academic hospital in Milan
Nguyen 2022 (23)	case-control (2006-2016)	<50	2557	Sweden	All	CRC	antibiotics	The Swedish National Board of Health and Welfare and Epidemiology Strengthened by histoPathology Reports in Sweden(ESPRESSO)
McDowell 2022 (24)	case-control(1999-2011)	<50	445	Scotland	All	CRC, colon cancer, rectal cancer	antibiotics	population-based, Primary Care Clinical Information Unit Research (PCCIUR) database
Li 2022 (25)	case-control(2003-2020)	<50	339	Germany	All	CRC, colon cancer, rectal cancer	obesity	population-based,Chancen der Verhütung durch Screening(DACHS)
Jin 2022 (26)	cohort (2009-2010)	<50	8320	South Korean	All	CRC	MetS, obesity	population-based,National Health Insurance Service (NHIS)
Pang 2022 (27)	cohort (2014-2019)	<50	621	Canada	All	Colorectal Adenomas	age, female, BMI, undergone a diagnostic colonoscopy	Hospital-based
Danial 2022 (28)	case-control(1999-2019)	20-50	13800	The United States	All	CRC	family history, primary malignant neoplasm of breast, IBD, alcohol consumption, smoking, obesity, diabetes, hyperlipidemia, Caucasians	population-based, commercial database (Explorys)
Archambault 2021 (29)	case-control (1990s- 2010s)	<50	3767	European	All	CRC, colon cancer, rectal cancer	Family history, red meat, processed meat, smoking, alcohol consumption, Sedentary, diabetes	population-based,the Colon Cancer Family Registry, the Colorectal Transdisciplinary study, and the Genetics and Epidemiology of Colorectal Cancer Consortium
Kim 2021 (30)	cohort (1991-2015)	25-42	111	The United States	F	CRC	vitamin D	Nurses’ Health Study II
Zheng 2021 (31)	cohort (1991-2011)	25-42	1153	The United States	F	adenoma, distal colon and rectum	Western diet	Nurses’ Health Study II
Yue 2021 (32)	cohort (1991-2015)	26-45	332	The United States	F	CRC	empirical dietary index for hyperinsulinemia (EDIH), empirical lifestyle index for hyperinsulinemia (ELIH), Hyperinsulinemia dietary, lifestyle patterns	Nurses’ Health Study II
Schumacher 2021 (33)	case-control(2008-2018)	15-49	1032	Kaiser Permanente Southern California (KPSC)	All	colorectal adenocarcinoma	obesity	population-based, Kaiser Permanente Southern California (KPSC)
Nguyen 2021 (34)	cohort (1991-2015)	25-42	2911	The United States	F	adenoma	sulfur microbial diet	Nurses’ Health Study II
Joh 2021 (35)	cohort (1999-2015)	25-42	4364	The United States	F	adenomas	High sugar, sugar-sweetened beverage	Nurses’ Health Study II
Chen 2021 (36)	case-control (2006-2015)	18-49	4673	The United States	All	CRC, colon cancer (proximal and distal), rectal cancer	MetS	MarketScan databases
Hur 2021 (37)	cohort (1991-2015)	25-42	109	The United States	F	CRC	sugar-sweetened beverage	Nurses’ Health Study II
Chang 2021 (38)	case-control (2018-2019)	20–49	175	Canada	All	CRC	family history, sedentary, sugar-sweetened beverage, Westernized dietary pattern	population-based, Ontario Cancer Registry (OCR)
Demb 2020 (39)	cohort (1999-2016)	18-49	47 800	The United States	All	CRC	iron-deficiency anaemia, haematochezia	Veterans Health Administration (VHA)
Dash 2020 (40)	cohort (1995-2013)	<50	113	The United States	F	CRC	Obesity	Black Women's Health Study
Low 2020 (13)	case-control(1999-2014)	18–49	651	The United States	All	CRC	male, smoking, non-aspirin users, lower BMIs, weight loss of 5 kg or more	Veteran’s Health Administration (VHA)
Gausman 2020 (41)	case-control(2011-2017)	18–49	269	New York City	All	CRC	male, IBD, family history, obesity, smoking, diabetes,	Hospital-based
L'Heureux 2019 (42)	case-control (2008-2013)	<51	8623	Taiwan	All	CRC, colon cancer, rectal cancer	hypothyroidism	population-based, Taiwanese National Health Insurance Research Database
Liu 2019 (43)	cohort (1991-2011)	25-42	114	The United States	F	CRC, colon cancer, rectal cancer	obese	Nurses’ Health Study II
Syed 2019 (44)	cohort (2012-2016)	25-49	5710	The United States	NR	CRC	male, Caucasian, abdominal pain, rectal pain, altered bowel function, rectal bleeding, weight loss, family history, gastrointestinal malignancy, polyps, smoking, alcohol consumption, presence of colitis, obesity	population-based, commercial database (Explorys)
Glover 2019 (15)	cohort (2013-2018)	20-39	1700	The United States	All	CRC	Caucasian, male, diabetes, smoking, obesity	population-based, commercial database (Explorys)
Nguyen 2018 (45)	cohort (1991-2011)	25–42	118	The United States	F	CRC, colon cancer, rectal cancer	Sedentary TV viewing time	Nurses’ Health Study II
Levi 2017 (46)	cohort (1967-2010)	16-19	1089	Israel	M	CRC	Overweight, obesity	Israeli National Cancer Registry (INCR) database
Kwak 2016 (21)	cohort (2011-2013)	20-39	497	Korea	All	colorectal adenoma	smoking, alcohol consumption	Hospital-based
Wu 2013 (47)	cohort (2004-2005)	< 50	36	Taiwan	All	CRC	the chronic kidney disease patients not undergoing dialysis	Health Insurance Database 2005
Søndergaard 2013 (48)	cohort (1978-2009)	<45	1789	Denmark	NR	CRC	low education	data from the Central Population Register
Rosato 2013 (49)	case-control (1985-2009)	<45	329	Italy and Switzerland	All	CRC	family history, alcohol, processed meat	Hospital-based
Ghadirian 1998 (50)	case-control (1989-1993)	<50	118	Canada	All	Colon cancer	ever married, family history, constipation, use of laxatives	Hospital-based
Negri 1998 (51)	case-control (1992-1996)	<45	145	Italy	All	CRC, colon cancer, rectal cancer	family history	Hospital-based
Fuchs 1994 (52)	cohort (1989-1992)	<50	13	The United States	F	CRC	family history	The Nurses' Health Study and The Health Professionals Follow-up Study.
St John 1993 (53)	case-control (1952-1985)	<45	82	Australia	All	CRC	family history	family medical hospital
Peter 1989 (54)	case-control (1974-1982)	<45	147	United States-LA County	M	CRC, stratified (right-sided, traverse/descending, sigmoid, rectum)	fumes, wood, metal dust, deep fried foods, barbecued, alcohol consumption, smoking, smoked meats	Hospital-based

CRC, colorectal cancer; MetS, metabolic syndrome; BMI, body mass index; F, female; M, male; IBD, inflammatory bowel disease.

### Study quality

The NOS was used to evaluate the quality of the studies. The included studies were of high quality, with scores ranging from 6 to 9 (out of 9), and an average score of 7.2 (**Table 2**). Funnel plot results indicated that there was some publication bias (**Supplementary Material** Funnel plot).

**Table 2:** Risk of bias and quality assessment.

Author	Case/ Representative	Representative/Nonexposed	Controls/Exposure	Definition/Outcome not present	Confounding	Exposure/Outcome	Method/Follow-up	Nonresponse/Adequate follow-up	Score
Puzzono 2022 (22)	*	*	*	*	–	*	*	*	7
Nguyen 2022 (23)	–	*	–	*	**	*	*	*	7
McDowell 2022 (24)	*	*	*	*	*	*	*	*	8
Li 2022 (25)	–	*	*	*	**	*	*	*	8
Jin 2022 (26)	*	*	*	*	**	*	*	*	9
Pang 2022 (27)	*	*	*	*	**	*	–	–	7
Danial 2022 (28)	*	*	*	*	–	–	*	*	6
Archambault 2021 (29)	*	*	*	*	*	*	*	*	8
Kim 2021 (30)	–	*	–	*	**	–	*	*	6
Zheng 2021 (31)	–	*	*	*	**	*	*	*	8
Yue 2021 (32)	–	*	*	*	**	*	*	*	8
Schumacher 2021 (33)	*	*	*	*	**	*	*	*	9
Nguyen 2021 (34)	–	*	*	*	**	*	*	*	8
Joh 2021 (35)	–	*	*	*	**	*	*	*	8
Chen 2021 (36)	*	*	*	*	**	*	*	*	9
Hur 2021 (37)	–	*	*	*	**	*	*	*	8
Chang 2021 (38)	–	*	*	*	**	*	–	*	7
Demb 2020 (39)	–	*	*	*	**	*	*	*	8
Dash 2020 (40)	–	*	–	*	**	*	*	*	7
Low 2020 (13)	*	–	*	*	*	–	*	*	6
Gausman 2020 (41)	*	*	–	*	*	–	*	*	6
L'Heureux 2019 (42)	*	*	–	*	*	–	*	*	6
Liu 2019 (43)	–	*	–	*	**	*	*	*	7
Syed 2019 (44)	*	*	–	*	**	–	*	*	7
Glover 2019 (15)	*	*	*	*	**	–	*	*	8
Nguyen 2018 (45)	–	*	*	*	**	*	*	*	8
Levi 2017 (46)	*	*	*	*	–	*	*	*	7
Kwak 2016 (21)	*	*	*	*	*	–	–	*	6
Wu 2013 (47)	*	*	*	*	–	*	–	*	6
Søndergaard 2013 (48)	*	*	*	*	–	*	*	*	7
Rosato 2013 (49)	*	*	–	*	**	*	*	*	8
Ghadirian 1998 (50)	*	*	*	*	–	*	*	–	6
Negri 1998 (51)	*	*	*	*	–	–	*	*	6
Fuchs 1994 (52)	–	*	–	*	**	*	*	*	7
St John 1993 (53)	*	*	–	*	–	*	*	*	6
Peter 1989 (54)	*	*	*	*	–	*	*	*	7

The NOS is a quality scale that evaluates studies based on 3 broad categories: selection (maximum of four stars); comparability (maximum of two stars); and exposure/outcome (maximum of three stars). *, one point; **, two points; -, no points.

### Demographics

**Figure 2:**
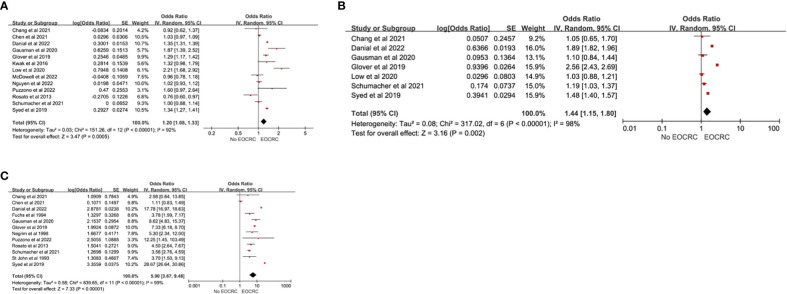
Demographic risk factors for EOCRC. **(A)** Male; **(B)** Caucasian; **(C)** Family History.

A total of 18 studies (13, 15, 21–24, 28, 33, 36, 38, 41, 44, 48–53) examined the association of demographic factors with the development of EOCRC. Male sex (pooled OR=1.20; 95% CI, 1.08-1.33) (13, 15, 21–24, 28, 33, 36, 38, 41, 44, 49), Caucasian race (pooled OR=1.44; 95% CI, 1.15-1.80) (13, 15, 28, 33, 38, 41, 44), and a family history of CRC (pooled OR=5.90; 95% CI, 3.67-9.48) (15, 22, 28, 33, 36, 38, 41, 44, 49, 51–53) were significantly associated with the development of EOCRC (**Figure 2**). There was significant heterogeneity in the effect estimates for all reported demographic factors (I^2^>60%). We did not find heterogeneity through sensitivity analysis. Based on the remaining studies, significant positive associations were found between a low education level (OR=1.64; 95%CI, 1.45-1.84) (48) and a family history of cancer (OR=11.66; 95%CI, 10.97-12.39) (44) and EOCRC. Ghadirian et al. found a negative association between married status (OR=0.58; 95%CI, 0.48-0.84) (50) and EOCRC.

### Chronic conditions

**Figure 3:**
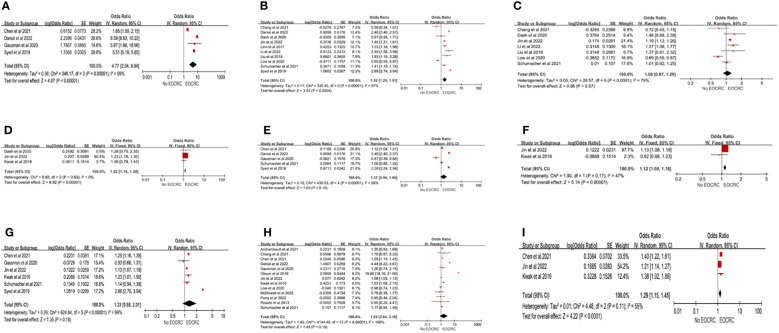
Chronic conditions risk factors for EOCRC. **(A)** IBD (Inflammatory bowel disease); **(B)** Obesity; **(C)** Overweight; **(D)** Abdominal Obesity; **(E)** Hyperlipidemia; **(F)** Triglycerides; **(G)** Hypertension; **(H)** Diabetes; **(I)** MetS (Metabolic Syndrome).

A total of 20 studies (13, 15, 21, 24–29, 33, 36, 38–44, 46, 49) examined the association between chronic conditions and EOCRC. IBD was significantly associated with the development of EOCRC (pooled OR=4.43; 95% CI, 4.05-4.84) (13, 15, 21, 24–29, 33, 36, 38–44, 46, 49), but the heterogeneity between studies was high (I^2^>60%). Three forms of obesity analysis were used in this study: (1) obesity (BMI≥30 kg/m^2^), (2) overweight (30 kg/m^2^>BMI>24.9 kg/m^2^), and (3) abdominal obesity. Obesity (pooled OR=1.52; 95%CI, 1.20-1.91) (13, 25, 26, 28, 33, 38, 40, 43, 44, 46) and abdominal obesity (pooled OR=1.22; 95%CI, 1.16-1.30) (21, 26, 40) showed significant associations with EOCRC, but overweight was not significantly associated with EOCRC (pooled OR=1.06; 95%CI, 0.87-1.29) (13, 25, 26, 33, 38, 40, 43). Heterogeneity was high in obesity (I^2 =^ 97%) and overweight (I^2 =^ 79%), but not in abdominal obesity (I^2 =^ 0%). In the sensitivity analysis for overweight, after excluding the study of Low et al. (13), the remaining studies were homogeneous (I^2 =^ 43%), and the results showed that overweight was significantly associated with the development of EOCRC (pooled OR=1.18; 95% CI, 1.12-1.25) (**Supplementary Material** Forest plot A). Liu et al. (43) found that for every 5-unit increase in BMI, the risk of EOCRC was 20% higher (RR=1.20; 95%CI, 1.05-1.38). We analyzed the associations of hyperlipidemia and triglycerides with EOCRC. Hyperlipidemia was not associated with EOCRC (pooled OR=1.37; 95% CI, 0.94-1.99) (28, 33, 36, 41, 44), but triglycerides were significantly associated with EOCRC (pooled OR=1.12; 95% CI, 1.08-1.18) (21, 26). For hyperlipidemia, the heterogeneity was high (I^2 =^ 99%), while the studies on triglycerides were homogeneous (I^2 =^ 47%). Six studies (21, 26, 33, 36, 41, 44) assessed the relationship between hypertension and EOCRC, and the results demonstrated that hypertension was not associated with the development of EOCRC (pooled OR=1.33; 95% CI, 0.88-2.01), but the heterogeneity was high (I^2 =^ 99%). After excluding the study by Syed et al. (44), the heterogeneity decreased to I^2 =^ 41%. The comprehensive results showed that hypertension was associated with EOCRC (pooled OR=1.16; 95% CI, 1.12-1.21) (**Supplementary Material** Forest plot B). The combined results of 13 studies (13, 15, 21, 24, 26–29, 33, 36, 38, 41, 49) showed that there was no significant difference between EOCRC and diabetes (pooled OR=1.63; 95%CI, 0.84-3.16), but high heterogeneity existed among studies (I^2 =^ 100%). Three studies (21, 26, 36) investigated the association between metabolic syndrome and EOCRC, and the results showed that metabolic syndrome was a high-risk factor for EOCRC (pooled OR=1.29; 95%CI, 1.15-1.45), but there was heterogeneity among studies (I^2 =^ 55%) (**Figure 3**). According to the remaining studies, a significantly higher risk of developing EOCRC was associated with chronic kidney disease in patients not undergoing dialysis (47), primary breast tumors (28), abdominal pain (44), iron-deficiency anemia (39), hematochezia (39), rectal pain (44), intestinal function changes (44), and weight loss (13). A significantly lower risk of developing EOCRC was associated with hyperthyroidism (42).

### Lifestyle behaviors and environmental factors

**Figure 4:**
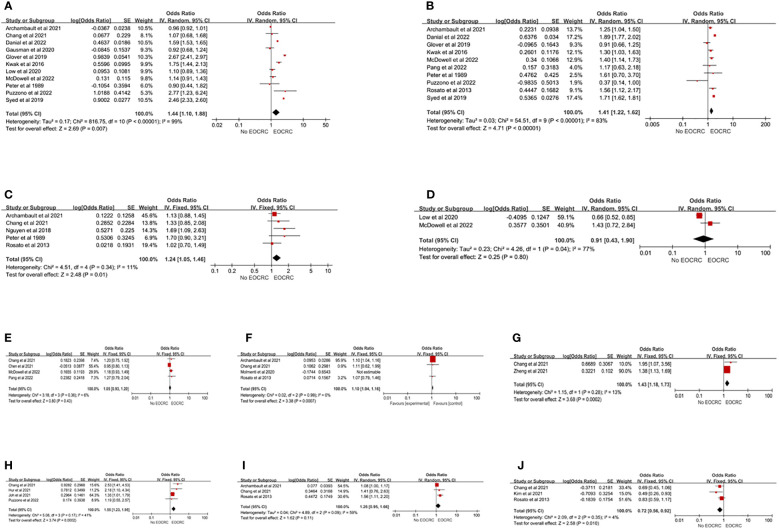
Lifestyle risk factors for EOCRC. **(A)** Smoking; **(B)** Alcohol Consumed; **(C)** Sedentary; **(D)** Aspirin; **(E)** NSAID; **(F)** Red Meat; **(G)** Westernized Dietary Pattern; **(H)** Sugar-Sweetened Beverage; **(I)** Processed meat; **(J)** Vitamin D.

Twenty-two studies (13, 15, 21–24, 26–32, 35–38, 41, 44, 45, 49, 54) evaluated the association between lifestyle or environmental factors and EOCRC. Smoking and drinking were identified through self-reporting. Self-reported smoking was divided into two categories (current or past smoker *vs*. never-smoker) or three categories (current smoker, past smoker, and never-smoker). This study conducted a meta-analysis in the form of two classifications (current or past smoker *vs* never-smoker). The results of 11 studies (13, 15, 21, 22, 24, 28, 29, 38, 41, 44, 54) showed that smoking increased the risk of EOCRC (pooled OR=1.44; 95% CI, 1.10-1.88), but the heterogeneity was high (I^2 =^ 99%). Each study classified alcohol consumption by the number of drinks per week or the amount of alcohol consumed. In this study, alcohol consumption was analyzed in the form of two categories (alcohol consumption *vs* never alcohol consumption). Ten studies (15, 21, 22, 24, 27–29, 44, 49, 54) showed that alcohol consumption increased the risk of EOCRC (pooled OR=1.41; 95% CI, 1.22-1.62), but the heterogeneity was high (I^2 =^ 83%). Five studies (29, 38, 45, 49, 54) illustrated that sedentary behaviors increased the incidence of EOCRC (pooled OR=1.24; 95% CI, 1.05-1.46), and there was homogeneity (I^2 =^ 11%). The results showed that aspirin (pooled OR=0.91; 95% CI, 0.43-1.90) (13, 24) and nonsteroidal anti-inflammatory drugs (NSAIDs) (pooled OR=1.05; 95% CI, 0.93-1.20) (24, 27, 36, 38) were not associated with the development of EOCRC. There was heterogeneity for aspirin (I^2 =^ 77%), while homogeneity was observed for NSAIDs (I^2 =^ 6%). Nguyen et al. (23) found that oral antibiotics were associated with the development of EOCRC (OR=1.18, 95% CI, 1.07, 1.29), but an inconsistent result was found by Chang et al. (38) (OR=0.78, 95% CI, 0.47, 1.30), while McDowell et al. (24) found that antibiotic consumption was associated with colon cancer (OR=1.49, 95% CI, 1.07, 2.07), but not with rectal cancer (OR=1.17, 95% CI, 0.75, 1.84). In terms of diet, red meat (pooled OR=1.10; 95% CI, 1.04-1.16) (29, 38, 49), Western dietary patterns (pooled OR=1.43; 95% CI, 1.18-1.73) (31, 38), and sugar-sweetened beverages (pooled OR=1.55; 95% CI, 1.23-1.95) (22, 35, 55, 56) were associated with the development of EOCRC. Processed meat was not associated with EOCRC (pooled OR=1.26; 95% CI, 0.95-1.66) (29, 38, 49), while vitamin D was a protective factor on EOCRC (pooled OR=0.72; 95% CI, 0.56-0.92) (30, 38, 49) (**Figure 4**). Heterogeneity only existed in processed meat (I^2 =^ 59%), but after excluding the study of Archambault et al. (29), the studies were homogeneous (I^2 =^ 0%). The results indicated that processed meat was associated with the development of EOCRC (pooled OR=1.53; 95% CI, 1.13-2.06) (**Supplementary Material** Forest plot C). Among the remaining studies, a significantly higher risk of developing EOCRC was associated with the empirical dietary index for hyperinsulinemia (EDIH) (32), the empirical lifestyle index for hyperinsulinemia (ELIH) (32), and exposure to dust (54), fumes (54), wood (54), metal dust (54), and sulfur microbial diet (34). A significantly lower risk of developing EOCRC was associated with vegetables, fruits, prudent diet, fish, and consumption of β-carotene, vitamin C, vitamin E, and folic acid (22, 49).

## Discussion

CRC is a global public health problem that seriously threatens human health. Although the overall incidence and mortality of CRC have tended to stabilize or decline in recent years, the incidence and mortality of EOCRC have shown an increasing trend, and EOCRC is usually diagnosed at later stages with a poor prognosis. Identifying risk factors for EOCRC is essential to reduce the growing burden of this disease.

In this systematic review and meta-analysis, we identified risk factors associated with demographics (male, Caucasian, family history), chronic conditions (IBD, obesity, overweight, abdominal obesity, hypertension, triglycerides, metabolic syndrome), and lifestyle or environmental factors (smoking, alcohol consumption, sedentary behaviors, sugar-sweetened beverages, red meat, processed meat, and Western dietary patterns). Although some of the factors (hyperlipidemia, diabetes) were statistically non-significant, they were suggestive and non-negligible risk factors. The study also found that vitamin D was a protective factor for EOCRC. Other potential risk factors included a family history of cancer, a low education level, chronic kidney disease, primary breast tumors, abdominal pain, intestinal function changes, iron-deficiency anemia, hematochezia, weight loss, exposure to dust, and diet-related factors.

Male sex, Caucasian race, and a family history of CRC were risk factors for EOCRC. The results were the same as those exhibited by O’Sullivan et al. (14). Individuals with a family history or CRC had a higher risk of CRC than the general population. Screening based on a family history of CRC has become an important screening strategy for early detection and prevention of EOCRC. Previous research also showed that people with a family history of other cancers had a higher risk of developing EOCRC. Therefore, future research also needs to examine whether a family history of other cancers before the age of 50 is related to an increased risk of EOCRC (28). Research on a family history of other cancers may guide more precise targeted screening strategies.

This study compared the effects of different degrees of obesity on EOCRC. We found that weight gain in young adults was associated with the risk of EOCRC. The results of this study were consistent with those of Li et al. (17). Since Glover et al. (15) did not define obesity and Kwak et al. (21) only compared individuals with BMI≥25 kg/m^2^ with the general population, these 2 studies were not included for the analysis in this study. Glover et al. (15) found that obesity was a risk factor for EOCRC (OR=1.82; 95% CI, 1.62-2.04), but Kwak et al. (21) suggested that obesity was not related to EOCRC. Liu et al. concluded that the risk of CRC increased by 20% for every 5 units of BMI increase. The results of this study also demonstrated that obese individuals had a higher risk of CRC than overweight individuals. The reason why obesity was related to EOCRC may be that obesity is involved in the occurrence and development of CRC by affecting metabolism and inflammatory factors, including insulin and insulin-like growth factors, sex hormones, and adipokines. Further research has found that obesity can promote the occurrence of CRC by affecting DNA methylation. In young mice, obesity can promote oxidation of long-chain fatty acids to increase the number of stem cells or stem cell-like cells in intestinal tissues (57). These findings also provide new clues to explain the mechanism by which obesity promotes EORC. Therefore, interventions to prevent obesity and strengthen obesity management in adolescents and young people are crucial to reduce the incidence of EOCRC.

No statistically significant difference was found between diabetes and hyperlipidemia and EOCRC in the present study, but this result was contrary to that reported by Breau et al. (55). Breau et al. assessed cross-sectional studies, while our study investigated case-control studies and cohort studies, and differences in the included literature may have influenced the results. In a Swedish national cohort study (56), more than 100,000 patients diagnosed with diabetes before the age of 50 were included. Diabetes was shown to be associated with a 1.9-fold increased risk of EOCRC. However, some studies found that diabetes was not associated with EOCRC (24). Given the increasing prevalence of diabetes in young people and the potential impact of CRC screening guidelines, more studies are required to confirm this relationship. Hyperlipidemia and hypertension are both associated with the development of CRC, but there is insufficient research on the relationship with EOCRC. More studies are needed in the future to assess whether hyperlipidemia is associated with the development of EOCRC, and to include triglyceride and cholesterol levels in the analysis at the same time, thereby identifying individuals at a high risk of EOCRC and benefiting from earlier screening.

Poor lifestyles were associated with EOCRC. Smoking has always been recognized as a risk factor for EOCRC, but the results of O’Sullivan et al. (14) suggested that smoking was not associated with the development of EOCRC, which was contrary to the results of this study. On the one hand, O’Sullivan et al. (14) comprehensively analyzed the results of cross-sectional studies, case-control studies, and cohort studies; on the other hand, only 5 studies were included for the meta-analysis, which may have a certain impact on the results of the study. Nicotine, an alkaloid in tobacco, can induce and promote the proliferation of colon cancer cells and the formation of tumor blood vessels (58). Diet is an important factor affecting EOCRC. The Western diet, which is high in saturated fat, rich in red meat, and low in fiber, has become a well-known risk factor for CRC. Adherence to a healthier diet, such as the Mediterranean diet, can help prevent CRC (59). Nguyen et al. (34) suggested that a high-sulfur microbial diet would increase the incidence of EOCRC (OR=1.13; 95% CI, 1.10-1.56). Microbes can metabolize sulfur in the diet to produce hydrogen sulfide, which is a gastrointestinal carcinogen. A healthy diet allows a more beneficial intestinal microbiota and may reduce the risk of CRC. Therefore, it is necessary to take public health measures against the unhealthy lifestyles and eating habits of young people to reduce the incidence of EOCRC.

Low et al. (13) found that aspirin had a protective effect on EOCRC, while McDowell et al. (24) reported that aspirin was not associated with the development of EOCRC. Considering the chemical protective potential of aspirin against CRC, the relationship between aspirin and EOCRC needs to be further evaluated. Studies on whether antibiotics play a role in EOCRC have varied. McDowell et al. (24) analyzed the effects of antibiotics on colon and rectal cancers separately and concluded that antibiotics only increased the risk of colon cancer. The correlation between different antibiotic types, different tumor sites and different ages needs further analysis. However, due to the limited number of studies included, whether these factors are related to the development of EOCRC remains to be further studied.

## Limitations

1) Most of the included studies used data from databases, with Danial et al. (28), Syed et al. (45), and Glover et al. (15) using the Explorys database, and Kim et al. (30), Zheng et al. (31), Yue et al. (32), Nguyen et al. (34), Joh et al. (35), Hur et al. (37), Liu et al. (44) using the Nurses’ Health Study II database, which made it possible for some data to be calculated multiple times. In addition, using the same database would lead to a decrease in the diversity of the population. 2) We only included studies published in English and may have omitted non-English studies. 3) We only searched for peer-reviewed journal studies and may have overlooked unpublished data. 4) Risk estimates for most risk factors were highly heterogeneous across studies. 5) Confounding factors were not controlled in some research results, which may lead to bias in the results. 6) Some of the results may have a publication bias.

## Conclusions

Smoking, drinking, sedentary behaviors, red meat, processed meat, sugary drinks, and Western dietary patterns were modifiable risk factors for EOCRC. IBD, obesity, high triglycerides, and hypertension were risk factors that are difficult to be changed by interventions. Although our study identified risk factors for EOCRC, further research is needed for validation and explore other aspects of EOCRC etiology to inform primary and secondary prevention measures. At the same time, our study provides the basis for the future construction of EOCRC risk prediction models to identify high-risk individuals and develop more targeted screening strategies, which in turn can better allocate resources to those most in need to cope with the global growth of EOCRC.

## Author contributions

All the authors contributed to the study design. QJ and XX screened abstracts and full-text articles for inclusion. QJ and PS appraised study quality. Any disagreements or uncertainties during the screening and quality appraisal process were referred to HH. QJ drafted the manuscript, which was reviewed, edited, and approved by HH. All authors contributed to the article and approved the submitted version.
