# Supplementary material for: Risk factors for early-onset colorectal cancer: systematic review and meta-analysis
Source: Front Oncol. 2023 May 5;13:1132306. doi: 10.3389/fonc.2023.1132306 (PMC10196487; doi:10.3389/fonc.2023.1132306)

Supplementary Material

**Risk factors for Early-Onset Colorectal Cancer：Systematic Review and Meta-Analysis**

**Hongmei Hua, Qiuping Jiang*, Xing Xu, Pan Sun**

*** Correspondence:** Qiuping Jiang: bottle@zju.edu.cn

# Supplementary Data

**1.1 search strategy**

**PubMed search strategy**

#1

(("Colorectal Neoplasms"[Mesh] OR "Colorectal Neoplasms, Hereditary Nonpolyposis"[Mesh]) OR "Colonic Neoplasms"[Mesh]) OR "Rectal Neoplasms"[Mesh]

#2

(((((((((((("colon tumor"[Title/Abstract]) OR ("colon malign*"[Title/Abstract])) OR ("colon canc*"[Title/Abstract])) OR ("rectum tumor"[Title/Abstract])) OR ("rectum malign*"[Title/Abstract])) OR ("rectum canc*"[Title/Abstract])) OR ("colorectal tumor"[Title/Abstract])) OR ("colorectal malign*"[Title/Abstract])) OR ("colorectal canc*"[Title/Abstract])) OR ("Colitis-Associated Neoplasms"[Title/Abstract])) OR ("Sigmoid Neoplasms"[Title/Abstract]))) OR ("CRC"[Title/Abstract])

#3

#1 OR #2

#4

"young onset" [Title/Abstract] OR "early onset" [Title/Abstract] OR "young adult*" [Title/Abstract] OR "early-onset" [Title/Abstract] OR "young-onset" [Title/Abstract] OR "adolescen*" [Title/Abstract] OR "under 50" [Title/Abstract] OR "younger than 50" [Title/Abstract] OR "15-49" [Title/Abstract] OR "under 40" [Title/Abstract] OR "younger than 40" [Title/Abstract] OR "under 30" [Title/Abstract] OR "younger than 30" [Title/Abstract] OR "under 20"[Title/Abstract] OR "younger than 20" [Title/Abstract]

#5

Risk Factors[Mesh]

#6

"risk factor"[Title/Abstract] OR "predictors" [Title/Abstract] OR "correlat*" [Title/Abstract] OR "influen*" [Title/Abstract] OR "inciden*" [Title/Abstract] OR "cause*"[Title/Abstract] OR associat [Title/Abstract] OR relat*[Title/Abstract] OR "related factor*" [Title/Abstract]

#7

#5 OR #6

#8

#3 AND #4 AND #7

**Embase search strategy**

#1

'colorectal tumor'/exp OR 'colon tumor'/exp OR 'rectum tumor'/exp

#2

'colorectal neoplasia':ab,ti OR 'colorectal neoplasm':ab,ti OR 'colorectal neoplasms':ab,ti OR 'colorectal tumour':ab,ti OR 'tumor, colorectal':ab,ti OR 'tumour, colorectal':ab,ti OR 'colon adenoma':ab,ti OR 'rectal neuroendocrine tumor':ab,ti OR 'rectum adenoma':ab,ti OR 'rectum cancer':ab,ti

#3

#1 OR #2

#4

'young adult':ab,ti OR 'early onset':ab,ti OR 'young onset':ab,ti OR adolescent:ab,ti OR 'under 50':ab,ti OR 'under the age of 50':ab,ti OR 'younger than 50':ab,ti OR '15 49':ab,ti OR 'under 40':ab,ti OR 'under the age of 40':ab,ti OR 'younger than 40':ab,ti OR 'under 30':ab,ti OR 'under the age of 30':ab,ti OR 'younger than 30':ab,ti OR 'under 20':ab,ti OR 'under the age of 20':ab,ti OR 'younger than 20':ab,ti

#5

'risk factor'/exp

#6

'risk factor':ab,ti OR predictors:ab,ti OR correlat*:ab,ti OR 'influenc* factors':ab,ti OR incidence:ab,ti OR associate*:ab,ti OR 'relat* factor*':ab,ti

#7

#5 OR #6

#8

#3 AND #4 AND #7

# Supplementary Figures

## Funnel plot

(A) Overweight


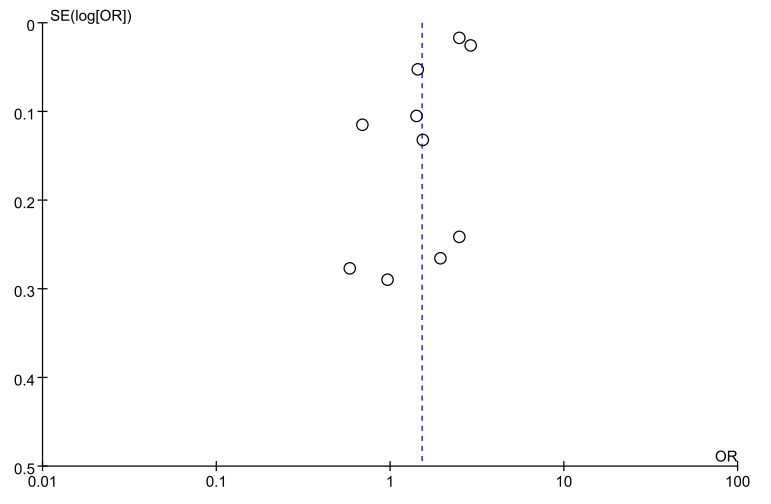


(B) Diabetes


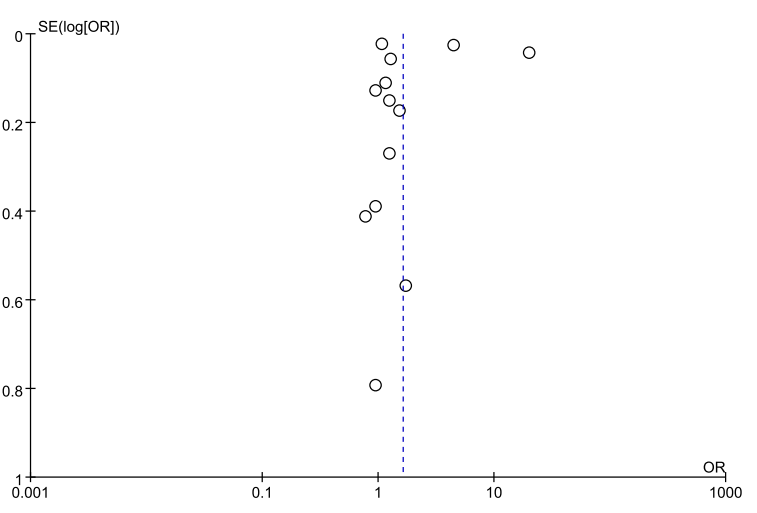


(C) Smoking


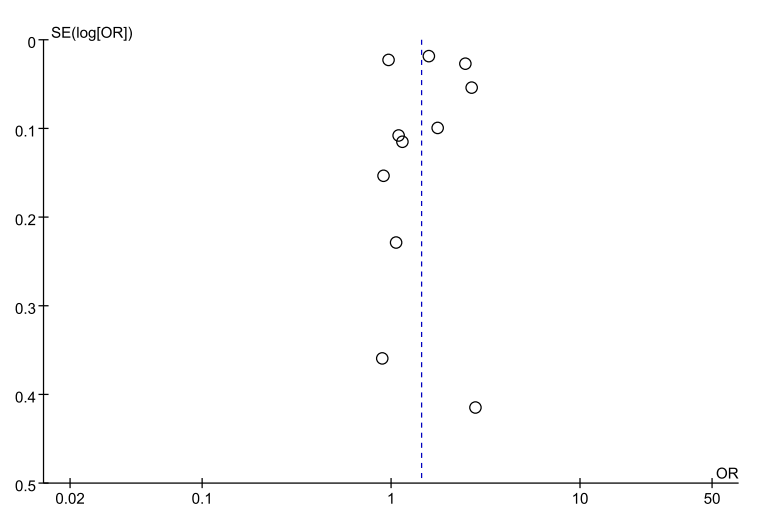


(D) Alcohol consumption


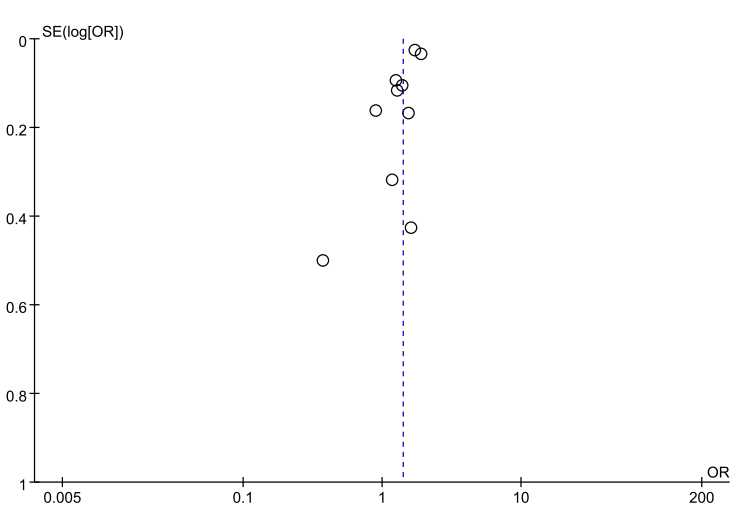


## 2.2 Forest plot

The results of heterogeneity can be removed after sensitivity analysis

A. Overweight


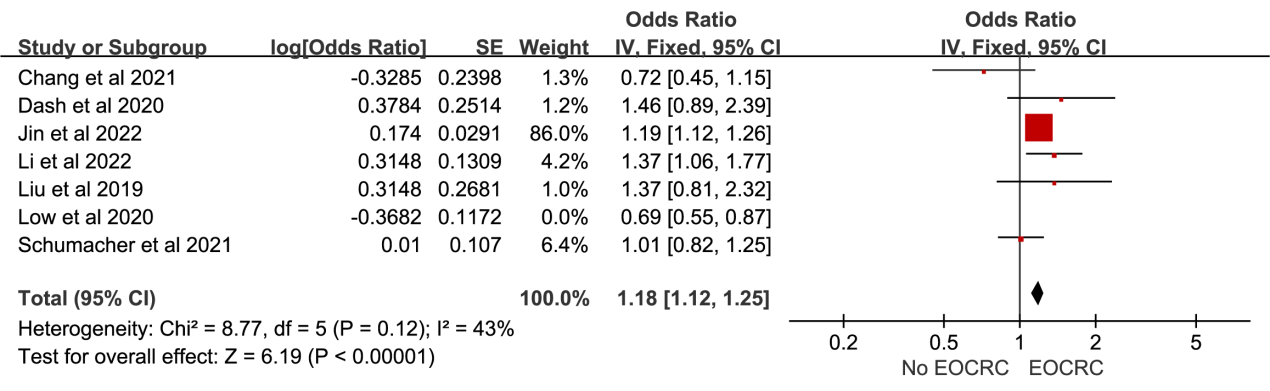


B. Hypertension


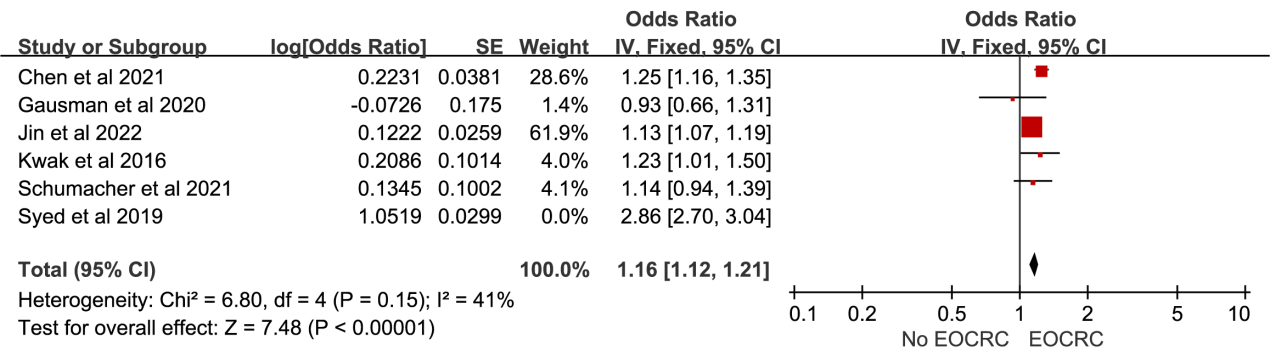


C. Processed meat


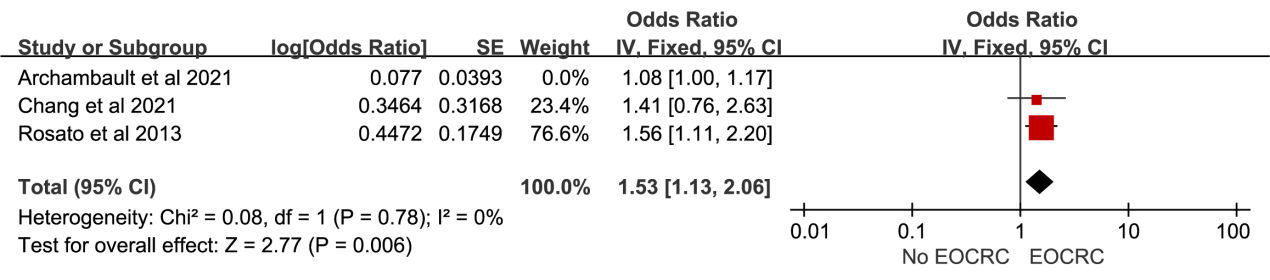

Supplement: Supplementary file 1 [file DataSheet_1.docx]
